# Mitral valve repair: moving towards a personalized ring

**DOI:** 10.1186/s13019-019-0926-7

**Published:** 2019-06-13

**Authors:** Antonios Pitsis, Timotheos Kelpis, Efstratios Theofilogiannakos, Nikolaos Tsotsolis, Harisios Boudoulas, Konstantinos Dean Boudoulas

**Affiliations:** 1 Department of Cardiac Surgery, St. Luke’s Hospital, Thessaloniki, Greece; 2 Department of Medicine, Division of Cardiovascular Medicine, The Ohio State University, Columbus, OH USA

**Keywords:** Mitral valve prolapse, Floppy mitral valve, Mitral regurgitation, Repair, Personalized ring

## Abstract

**Background:**

Mitral valve repair with the use of an annuloplasty ring is the procedure of choice in patients with significant mitral regurgitation (MR) due to floppy mitral valve (FMV)/mitral valve prolapse (MVP). The mitral annular size, shape and motion may vary substantially among patients and thus, commercially available rings may not be suitable for each individual patient.

**Methods:**

A “personalized ring” (PR) was easily constructed in the operating room using a Dacron sheet and titanium ligating clips to custom fit to each individual mitral annulus shape and size. There were 127 patients with severe MR due to FMV/MVP that underwent mitral valve repair surgery; 58 patients received a PR and 69 patients received a commercial Carpentier-Edwards Physio II ring. The patient records were retrospectively analysed.

**Results:**

There were no surgical deaths. In-hospital length-of-stay and blood transfusions were not statistically different between the two groups. Mitral valve area was greater (*p* < 0.05) in the PR group (3.78 ± 0.22) compared to the Physio II ring group (3.13 ± 0.21). Mitral annular area changed from systole to diastole by 14.35% ± 3.28% in the PR group and did not change in the Physio II ring group (*p* < 0.05). Systolic anterior motion (SAM) of the mitral valve occurred in 2 patients with the Physio II ring and no patients with the PR. Up to 8 years follow-up, all patients in both groups were alive with NYHA functional class I-II symptoms and mild or less MR.

**Conclusions:**

The PR is suitable for all patients with significant MR due to FMV/MVP who require MV repair. The precise fit of the PR to the mitral annulus better preserves valve area and sphincter function of the mitral annulus, prevents SAM and provides excellent short and long-term results.

## Key question

Whether mitral valve repair with a personalized ring is better in preserving mitral valve function compared to the Physio II ring.

## Key findings

The personalized ring preserves mitral annular sphincter function, prevents systolic anterior motion (SAM) of the mitral valve and is associated with greater mitral valve area.

## Take home message

Mitral valve repair with the personalized ring better preserves physiologic function of mitral valve compared to the Physio II ring.

## Introduction

Mitral valve surgery is indicated in almost all symptomatic patients with significant mitral regurgitation (MR) due to floppy mitral valve (FMV)/mitral valve prolapse (MVP). When surgery is performed in these patients, mitral valve repair is superior to mitral valve replacement, as it preserves left ventricular function and eliminates the potential inherent adverse effects related to a prosthetic valve including anticoagulation therapy, thrombosis, endocarditis, valve malfunction, noise due to mechanical prosthesis, among others. In certain circumstances, surgery also should be considered in asymptomatic patients, particularly if the likelihood of mitral valve repair is high, operative risk low, and surgery is performed in an experienced mitral centre [1–6].

The use of a mitral annuloplasty ring in mitral valve repair surgery, among others, remodels the annulus allowing for equal distribution of stress across the mitral leaflet scallops, an important factor for the durability of repair. Ideally, the mitral annuloplasty should result in the preservation of the dynamic motion of the annulus of the mitral valve.

Although the use of an annuloplasty ring has been a standard practice in mitral valve repair, the choice of the actual type of ring or band is driven by a surgeon’s preference and/or familiarity with the device [7–9]. Over the last two decades at St. Luke’s Hospital, Thessaloniki, Greece, more than fifteen hundred surgical mitral valve repairs in patients with MR due to FMV/MVP were performed. Over this long period of time and extensive experience, it became apparent that commercially available full rings did not actually restore physiological annular function. In order to better serve the need of the individual patient, a new “personalized” annuloplasty ring was developed. The accumulative experience of the “personalized ring” in patients with MR due to FMV/MVP is presented.

## Methods

### Mitral “personalized ring”

**Fig. 1:**
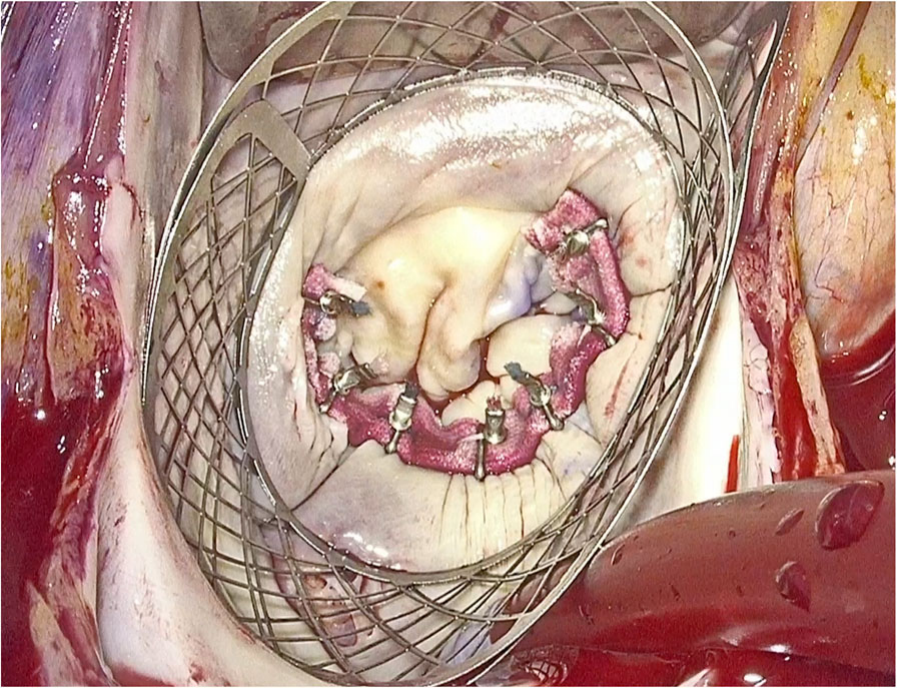
“Personalized ring” used for posterior mitral annuloplasty. The sutures are tied with the COR-KNOT device (CSI Solutions, USA)

**Fig. 2:**
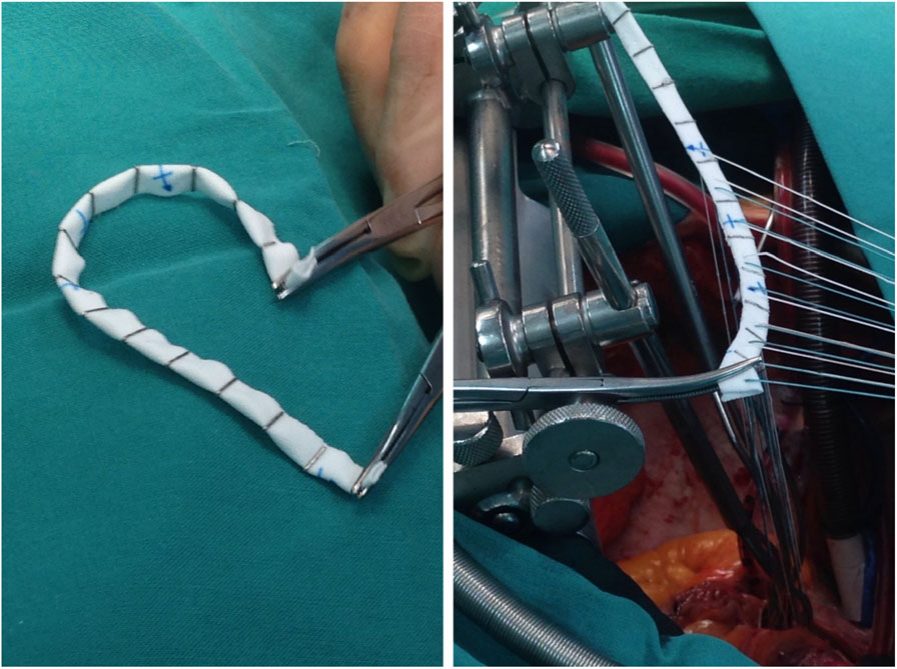
Preparation and insertion of a “personalized ring” during mitral valve repair performed through median sternotomy

The mitral “personalized ring” was constructed intra-operatively. A Dacron sheet (Bard Sauvage Filamentous Knitted Polyester Fabric) of 12 cm × 2 cm was cut and folded upon itself along its longitudinal axis to form a double layer band. In order to hold the band in position, titanium ligating clips where placed serially at 1 cm apart (Teleflex Weck Hemoclip Medium Titanium Ligating Clips) (Figs. 1 and 2). The length of the “personalized ring” was always adjusted to the size of the mitral annulus of each individual patient in such a way that the number of annuloplasty sutures used corresponded to the number of titanium clips [2]. The two needles connected to the annuloplasty sutures were passed on either side of the titanium clips securing the sutures over the clips; this created a cross type of effect stabilizing that particular segment of the annulus, thus allowing the remaining segments of the annulus to move freely in the area between the sutures (like the links of a chain). Our technique is presented in a video on CTSNet [3], which can be viewed on https://youtu.be/3EExhS6vkI0.

### Patient population

Between January 2010 and December 2013, 127 consecutive symptomatic patients with significant MR due to FMV/MVP who underwent mitral valve repair at St. Luke’s Hospital, Thessaloniki, Greece, were studied. Symptoms were due to MR and all patients were New York Heart Association (NYHA) functional class III or IV. An apical holosystolic murmur, consistent with significant MR, was present in all patients. Pre-operative transthoracic and transoesophageal (TOE) echocardiograms were performed in all patients, which demonstrated FMV/MVP and significant MR. Cardiac catheterization with left ventriculography and coronary arteriography were also performed prior to surgery. Significant MR was also documented by left ventriculography. Significant coronary artery disease (≥ 70% stenosis in at least one major coronary artery) was present in 15 patients. Patients with MR due to rheumatic valve disease, endocarditis and coronary artery disease with left ventricular dysfunction were excluded. In addition, patients who required redo cardiac surgery were excluded.

From the 127 patients, the mitral “personalized ring” was used in 58 patients and the commercially available semi-flexible annuloplasty ring (Carpentier-Edwards Physio II) was used in 69 patients [1, 2, 8, 9]. The selection between the two rings was made purely according to chronologic order (from Physio II to “personalized ring”) without overlap. The median follow-up period for the Physio II ring was 86 months (range 78–96 months) and for the “personalized ring” was 72 months (range 61–78 months). Age, gender, NYHA functional class, severity of MR, type of prolapse/flail (posterior, anterior, or bileaflet), and echocardiographic data were not statistically different between the two groups (Tables 1, 2 and 3). The study was approved by the Institutional Review Board of St. Luke’s Hospital Thessaloniki Greece, and informed consent was obtained from all patients.

**Table 1:** Clinical data in patients studied

	Personalized ring (*n* = 58)	Physio II ring (*n* = 69)	*P*-value
Age	59.3 ± 15.2	57.2 ± 12.5	0.38
Female	13 (22.4%)	13 (18.8%)	0.61
NYHA, FC	3.2 ± 0.5	3.2 ± 0.4	0.65
Atrial fibrillation	8 (13.8%)	8 (11.6%)	0.70
CAD	9 (15.5%)	6 (8.7%)	0.13

*CAD* Coronary artery disease, *FC* Functional class, *NYHA* New York Heart Association

**Table 2:** Preoperative echocardiographic data in patients studied

	Personalized ring (*n* = 58)	Physio II ring (*n* = 69)	*P*-value
Degree of MR	3.5 ± 0.5	3.6 ± 0.4	0.19
LVESD (cm)	3.7 ± 0.5	3.6 ± 0.4	0.33
LVEDD (cm)	5.6 ± 0.7	5.2 ± 0.5	0.24
LA diameter (cm)	4.6 ± 0.6	4.3 ± 0.7	0.25
EF	62 ± 6%	60 ± 6%	0.31
PASP (mmHg)	36 ± 8	38 ± 9	0.29
Vena Contracta (mm)	6.1 ± 0.7	6.1 ± 0.6	0.35

*EF* Ejection fraction, *LA* Left atrial, *LVEDD* Left ventricular end diastolic diameter, *LVESD* Left ventricular end systolic diameter, *MR* Mitral regurgitation, *PASP* Pulmonary artery systolic pressure

**Table 3:** Type of prolapse/flail

	Personalized ring (*n* = 58)	Physio II ring (*n* = 69)	*P*-value
Bileaflet prolapse/flail	7/4	9/7	0.89
Anterior prolapse/flail	4/3	3/1	0.81
Posterior prolapse/flail	47/44	57/52	0.81

### Surgical procedure

Surgery was undertaken through a median sternotomy. Cardiopulmonary bypass (CPB) was established via aorto-bicaval cannulation. Cardiac arrest and cardio-protection were achieved through warm-blood cardioplegia. The mitral valve was exposed via the superior septal approach. FMV was also confirmed in all patients in the operating room by direct inspection from the surgeon who performed the procedure; all mitral valve repairs were performed by the same surgeon (AP) who had the experience of more than a thousand mitral valve repairs prior to the study period. Following mitral valve leaflet repair, insertion of either the “personalized ring” or the commercially available Physio II ring was performed. The same surgical techniques were used throughout the study period to treat posterior (quadrangular or trigonal resection), anterior (synthetic chordae) or bileaflet prolapse (resection of the posterior leaflet and synthetic chordae for the anterior, or edge-to-edge repair in one case of bileaflet prolapse in each group). If resections were deemed necessary, special care was given to minimize the amount of posterior leaflet tissue resected.

The mitral “personalized ring” was inserted using mattress sutures placed into the posterior annulus and extending anteriorly to the trigones (Fig. 1). Seven to eleven mattress, non-pledgeted 2–0 sutures (Ethibond; Ethicon, Inc., Somerville, NJ) were most commonly used. The length and depth of the needle bite of the annuloplasty suture was 8–12 mm and 3–6 mm respectively at a right angle to the mitral annulus. Special care was given to stay exactly at the annular level and away from the atrial wall. The length of the “personalized ring” was dictated by the number of stiches used for the annuloplasty [3]. The “personalized ring” was inserted in the portion of the mitral annulus that was affected by the disease process. This approach was important in order to restore a close to normal geometry and function of the mitral valve. The area of the mitral annulus corresponding to the prolapsed or flail segment of the posterior leaflet required the maximum correction, thus this segment of the annulus was reduced with suture placement by as much as 50%. The commissural areas that play an important role in maintaining the 3:4 vertical to transverse ratio of the mitral annulus were assessed individually and accordingly corrected if needed. Likewise, the trigonal areas of the mitral annulus, which are important for maintaining a strong attachment of the posterior portion of mitral annulus to the fibrous skeleton of the heart, were individually assessed and corrected as needed. Segments of the annulus that were calcified were excluded from annular repair; among others, this approach decreases the risk of calcium dislodging that can result in embolization.

Sizing and implantation of the commercially Physio II ring was performed in the standard fashion as described previously [9].

To evaluate mitral valve function after mitral valve repair and before closure of the chest, while the patient was still on cardiopulmonary bypass, a TOE was performed. Arterial pressure and heart rate were altered with pharmacologic agents to mimic pressures and heart rates that are seen during usual daily activities.

In addition to mitral valve repair, 14 patients underwent coronary artery bypass surgery, 11 patients underwent tricuspid valve repair, 6 patients underwent aortic valve replacement, and 1 patient underwent atrial septal defect closure (Table 3).

### Follow-up

After discharge, patients were followed every 6 months for up to 8 years. At each follow-up visit, medical history, NYHA functional class, physical examination and transthoracic echocardiography were obtained in all patients. When the degree of MR was mild or less, and the functional status of the patient was good (functional class II or less), detail echocardiographic parameters were not measured after the 6-month follow-up.

### Statistical analysis

The data are presented in mean values ±1 standard deviation, except if indicated otherwise. The Student’s t-test was used for differences between means of continuous variables, while the chi-square or Fisher’s exact test were used to define differences between categorical variables. A *P*-value < 0.05 was considered statistically significant. SPSS 21.0 (IBM Inc., Armonk, NY) and JMP 11.0 (SAS Inst., Cary, NC) software were used for data analysis.

## Results

Demographic and echocardiographic parameters prior to surgery are shown in Table 1; the degree of MR, left ventricular ejection fraction, left ventricular end-diastolic and systolic diameters, left atrial diameters, and NYHA functional class were not statistically different between the two groups. Patients who had other surgical procedures, in addition to mitral valve repair, are shown in Table 4; however, these were a small number of patients to make any meaningful conclusions.

**Table 4:** Number of patients who had other surgical procedures in addition to mitral valve repair

	Personalized Ring (*n* = 58)	Physio II Ring (*n* = 69)
Aortic valve replacement	5 (8.6%)	1 (1.5%)
Tricuspid valve replacement	9 (15.5%)	2 (2.9%)
Coronary artery bypass	9 (15.5%)	5 (7.2%)
Atrial septal defect closure	1 (1.7%)	0 (0%)

Two patients from the Physio II group developed systolic anterior motion (SAM) of the mitral valve. The diagnosis of SAM in those patients was made intra-operatively after coming off CPB and was corrected with the use of artificial cordae to the anterior leaflet.

The mean blood loss was 6.9 ml/kg/24 h for patients who received the “personalized ring” and 7.2 ml/kg/24 h for those who received the Physio II ring. In the “personalized ring” group, 21 (36.2%) patients received one unit of blood and 14 (24.2%) received two units of blood, while 23 (39.6%) patients did not require a blood transfusion. In Physio II ring group, 24 (34.7%) patients received one unit of blood and 19 (27.6%) received two units of blood, while 26 (37.7%) patients did not require a blood transfusion. Hospital mortality was 0% in both groups. The median intensive care unit (ICU) length-of-stay (LOS) was 2 days for both groups. The median hospital LOS was 7 days for patients who received the “personalized ring” and 8 days for patients who received the Physio II ring. There was no statistically significant difference between the two groups in respect to blood loss, blood transfusion, ICU LOS and hospital LOS.

The echocardiographic parameters at discharge were also similar between the two groups. The degree of MR was mild or less (≤ 1+) in all patients in both groups. Basic hematologic and biochemical parameters at the time of discharge were not statistically different between the two groups.

### Follow-up

Follow-up was performed every 6 months for up to 8 years. All patients in both groups, during the entire follow-up period, were NYHA functional class I or I I and the degree of MR was mild or less than mild (≤ 1+). The six -months follow-up echocardiographic parameters are shown in Table 5. Since all patients had less than mild MR, as defined by echocardiography, and all were in good functional status (functional class I or II), detail echocardiographic parameters were not measured after the 6 months follow-up period.

**Table 5:** New York Heart Association Functional Class (NYHA FC) and echocardiographic parameters during the 6 months follow-up

	Personalized Ring (*n* = 58)	Physio II Ring (*n* = 69)	*P*-value
NYHA FC	1.2 ± 0.4	1.1 ± 0.3	0.40
LVESD	3.5 ± 0.5	3.4 ± 0.3	0.28
LVEDD	5.2 ± 0.6	4.9 ± 0.5	0.16
LA Diameter	4.5 ± 0.6	4.3 ± 0.7	0.24

*LA* Left atrial, *LVEDD* Left ventricular end-diastolic diameter, *LVESD* Left ventricular end systolic diameter

**Fig. 3:**
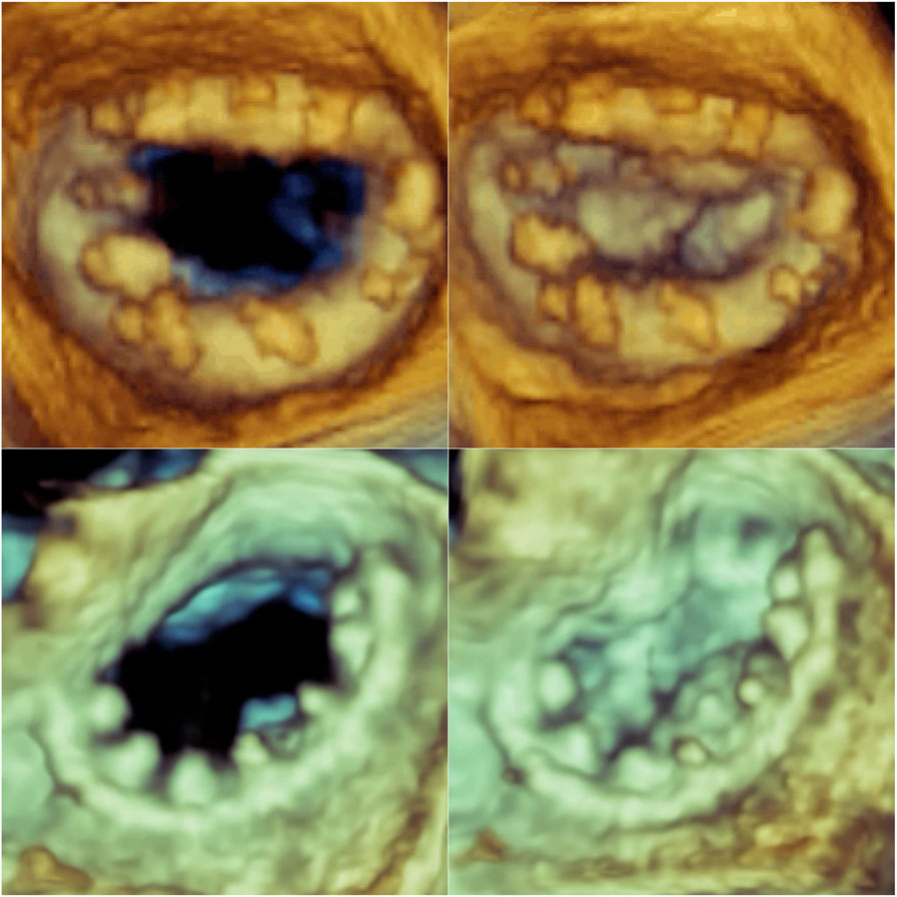
Commercial Physio II ring (upper panel) and the “personalized ring” (lower panel) are shown during diastole (left) and systole (right). Note that the mitral area in a patient with the “personalized ring” is dynamically changing from diastole to systole by 14%, while this area remains unchanged during the cardiac cycle in a patient with the commercial Physio II ring

The change in mitral valve annular area from diastole to systole was evaluated in 15 randomly selected patients in each group during the 6-month follow-up visit using TOE (Phillips Epiq 7C); every third patient that presented to the clinic for follow-up was selected for this analysis. The mitral valve area changed from systole to diastole by 10 to 20% (mean 14.35 ± 3.28%) in patients who received the “personalized ring”, while there was no change seen in those who receive the Physio II ring (*p* < 0.05) (Fig. 3). Thus, the sphincter function of the mitral annulus was preserved in patients who received the “personalized ring”, but not in those with the commercial Physio II ring. In the same randomly selected patients from each group, the mitral valve area in those who received the Physio II ring was smaller compared to those who received the “personalized ring” (3.13 ± 0.21 versus 3.78 ± 0.22, *p* < 0.05).

## Discussion

The results of this study demonstrated that the use of the “personalized ring” for mitral annuloplasty is suitable for all patients with MR due to FMV/MVP who required mitral valve repair. Mitral annuloplasty using the “personalized ring” provides excellent long-term results up to 8 years. The “personalizedd ring” can easily be constructed in a relatively short period of time in the operative room and can be adjusted to the size of the annulus for each individual patient with accuracy of less than 1 mm.

The precise fit of the “personalized ring” provides excellent support of the mitral annulus, which is important to prevent annular dilatation and paravalvular leak. In order to maintain the physiological sphincter function of the mitral valve annulus and the saddle shape of the mitral valve, mitral annuloplasty using the “personalized ring” was placed predominantly in the affected areas and not on the entire mitral annulus. In patients who received the “personalized ring”, the mitral annular area changed from systole to diastole by 14.35 ± 3.28% and thus, preserving the sphincter function of the mitral annulus. In contrast, the complete Physio II ring does not allow the mitral annulus to change in size during the cardiac cycle, thus the normal sphincter function of the mitral annulus is lost and the normal saddle shape of the mitral valve is converted to a flatter one. In addition, the mitral valve area in patients who had the “personalized ring” was greater compared to those who had the Physio II ring. This may have some impact on the long-term results and in functional capacity, particularly during strenuous exercise. Further, the precise adjustment of the “personalized ring” to the mitral annulus preserves the normal motion of the mitral valve and prevents abnormal SAM of the mitral valve, a known complication of this type surgery. In this study, two patients who received the commercial ring had SAM, while SAM was not seen in any of the patients who had the “personalized ring”. Moreover, the “personalized ring” is inexpensive compared to commercially available rings (less than 20 euros versus at least 1000 euros) and thus, cost effective.

Over the last two decades at St Luke’s Hospital more than 1500 surgical mitral valve repairs were performed by the same surgeon (AP). With this extensive experience, it became obvious that commercially available complete rings have the limitation of fixing the host annulus to a certain shape and forcing the annulus to conform to the size of the ring, which is a major disadvantage. In contrast, the “personalized ring” is flexible and is made to conform to the annulus of the individual patient allowing for the preservation of mitral annular function, a larger mitral valve area, and the elimination of SAM.

Our experience with the “personalized ring” is not limited to the 58 patients that were compared to the commercial Physio II ring, but also to an additional 330 patients of whom 200 were operated through a median sternotomy and 130 operated by a fully endoscopic procedure (procedure that has currently become our first line treatment for mitral repair) [3]. Importantly, SAM of the mitral valve was not seen in any of these patients (*n* = 388) who had the “personalized ring”, while present in 2 patients (*n* = 69) who had the Physio II ring (*P* = 0.031; Fisher’s exact test). During the follow-up period (every 6 months up to 8 years), all patients were NYHA functional class I or II and the degree of MR by echocardiography was mild or less (≤1+) in all 330 patients who received the “personalized ring”.

### Floppy mitral valve-mitral valve prolapse

“Variability is the law of life, and as no two faces are the same, so no two bodies are alike, and no two individuals react alike and behave alike under the abnormal conditions which we know as disease” – Sir William Osler

FMV/MVP is a common mitral valve disorder with a wide spectrum of structural and functional abnormalities from mild to severe [1, 2, 9–11, 13]. The term FMV comes from surgical and pathological studies and refers to the intrinsic pathological changes resulting in expansion of the area of mitral valve leaflets, elongation of chordae tendineae and mitral annular dilatation [1–15]. Mitral annular dilatation is a consistent finding in patients with FMV/MVP that ranges from mild to severe and can also be present in patients with minimal MR; this indicates that dilatation of the mitral annulus is primarily intrinsic and not a secondary abnormality due to left atrial and/or left ventricular dilatation in patients with significant MR from FMV/MVP [12–17]. Mitral annular dilatation is usually greater in patients with FMV/MVP associated with a heritable connective tissue disorder such as Marfan syndrome, Ehlers-Danlos syndrome, among others [11–15].

During the long “natural” history of the disease, certain patients with FMV/MVP and significant MR will eventually require mitral valve surgery (valve reconstruction or replacement). After reconstructive surgery, these patients usually have a long and productive life. In fact, reconstructive surgery in patients with FMV/MVP and MR has become part of the natural history in several of these patients [1, 2, 11]. Due to the significant variability in size of the mitral annulus, it is obvious that one ring size cannot be used for all patients. As we have started practicing personalized medicine in several other areas, the use of the “personalized ring” for mitral valve reconstructive surgery provides a rational approach for this purpose, the time of which has already past [18]. The results of the present study strongly support this notion. Further, the personalized ring can be used in other causes of MR such as functional MR and in patients with tricuspid regurgitation when surgical correction is indicated. Moreover, the personalized ring will be better suitable in patients with a large mitral annulus as is the case in patients with heritable connective tissue disorders such as the Marfan syndrome.

### Limitations

This is a retrospective study of a single center – single surgeon experience. Despite this limitation, however, based in our experience, it can be stated that the personalized ring is superior to commercially available rings. Multi-center randomized studies comparing commercially available rings to the “personalized ring” for the treatment of MR due to FMV/MVP and/or national and international registries would be helpful to definitely prove this thesis.

## Conclusions

A “personalized ring” is introduced for mitral valve repair in patients with FMV/MVP and significant MR. In contrast to the commercially available complete rings, the “personalized ring” preserves the saddle shape of the mitral valve and the sphincter function of the mitral annulus, and offers a larger mitral valve area. Further, the “personalized ring” prevents SAM, a know complication of this type of surgery. In addition, the “personalized ring” is much less expensive and thus, cost-effective.

## Data Availability

The supporting data could immediately become available to the reviewers and editors of the journal.

## Abbreviations

CTSNet: Cardio-Thoracic Surgical Network
FMV/MVP: Floppy mitral valve / mitral valve prolapse
ICU: Intensive care unit
LOS: Length of stay
MR: Mitral regurgitation
NYHA: New York Heart Association
SAM: Systolic anterior motion
TOE: Transoesophageal echocardiography
